# Primary cardiac lymphoma presenting with cardiac tamponade and complete heart block: case report

**DOI:** 10.1093/ehjcr/ytad635

**Published:** 2023-12-23

**Authors:** Ruth Kelleher, Brian Healey Bird, Tríona Hayes, Crochan J O’Sullivan

**Affiliations:** Department of Radiation Oncology, Cork University Hospital, Wilton Road, T12 DC4A Cork, Ireland; Department of Oncology, Bon Secours Hospital Cork, College Road, Cork, Ireland; Department of Medicine, University College Cork, College Road, Cork, Ireland; Department of Pathology, Bon Secours Hospital Cork, College Road, Cork, Ireland; Department of Medicine, University College Cork, College Road, Cork, Ireland; Department of Cardiology, Bon Secours Hospital Cork, College Road, Cork, Ireland

**Keywords:** Primary cardiac lymphoma, Cardiac tamponade, Complete heart block, Multimodality cardiac imaging, Endomyocardial biopsy, Case report

## Abstract

**Background:**

Primary cardiac lymphoma (PCL) is rare and its presentation can be variable. Thorough workup of suspected PCL or other cardiac tumours typically incorporates a range of imaging techniques and laboratory investigations but ultimately diagnosis is confirmed by histological analysis of myocardial tissue.

**Case Summary:**

An 80-year-old Caucasian female presented with complete heart block and symptomatic cardiac tamponade. A pericardiocentesis was performed and a dual-chamber permanent pacemaker was implanted for the management of her complete heart block. Subsequently, a right atrial mass was discovered on imaging and the patient underwent endomyocardial biopsy of the mass. Histological analysis of the sample confirmed a primary cardiac lymphoma. The patient opted to forgo treatment with chemotherapy and died from her disease 1 month later.

**Discussion:**

Cardiac arrhythmias can occur in PCL due to infiltration of conduction pathways. Characterization of cardiac masses on various imaging modalities and laboratory tests guides diagnosis. Tissue diagnosis is required to confirm PCL. The location of a cardiac mass may present technical challenges when undertaking a biopsy, so the best approach to tissue sampling should be considered on an individual basis. Without treatment survival is less than one month but with modern chemoimmunotherapy five-year survival may exceed 50%.

**Learning points**

- Primary cardiac lymphomas (PCL) can present a diagnostic challenge due to their rarity and variable presentations.
- The use of multiple imaging modalities is integral to the diagnostic process for primary cardiac lymphomas.
- Using a steerable catheter sheath during endomyocardial biopsy can optimize the positioning of the bioptome and aid in the overall ease of the procedure.
- Modern chemoimmunotherapy significantly improves 5-year overall survival.

## Introduction

Primary cardiac lymphomas (PCLs) are a rare diagnosis, accounting for only 1% of all cardiac tumours.^1^ Most primary tumours of the heart are benign (∼75%).^2^ Of the remaining small proportion of malignant primary cardiac tumours, sarcoma, and lymphoma are the most common. Literature suggests that PCL occurrence is more common in immunocompromised patients.^3,4^ The presentation of PCL can be variable, often reflecting the location, rate of growth, and size of the infiltrating disease within the heart. Individuals can present with obstructive symptoms secondary to the cardiac tumour, arrythmias related to the infiltration of conduction pathways, pericardial effusions, and tamponade; or embolization of tumours.

The optimal investigative process for a cardiac mass or suspected primary cardiac malignancy may include the use of such multimodality imaging as echocardiography, computed tomography (CT), cardiac magnetic resonance imaging (CMR), or Positron emission tomography (PET). This is outlined in the European Society of Cardiology (ESC) cardio-oncology guidelines 2022. The guidelines also stipulate that a histological diagnosis is required in cases of B-cell lymphomas.^5^

We report the case of a patient who presented with complete heart block and cardiac tamponade. We highlight how a combination of multimodality imaging and histological analysis of an endomyocardial tissue sample was used to establish a final diagnosis of PCL.

## Summary figure

**Figure:**
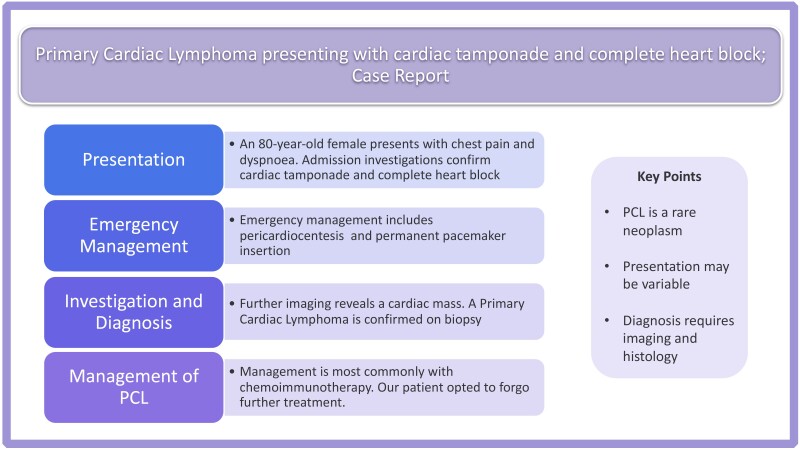


## Case summary

An 80-year-old Caucasian female presented with acute chest pain and one week of progressive dyspnoea on exertion on a 6-month background history of fatigue. Her medical history was notable for hyperlipidaemia, hypertension, and depression. Her medications included a Selective Serotonin Reuptake Inhibitor, a statin, an anti-muscarinic, a proton pump inhibitor, and calcium supplementation. There was no history of autoimmune disease, malignancy, tuberculosis, or HIV.

On admission, physical examination revealed signs consistent with heart failure (i.e. bilateral peripheral oedema, bibasal crepitations on auscultation of lungs, and mild epigastric tenderness). Initial blood pressure readings were within normal range. Heart sounds were normal with no added sounds. There was no jugular venous distention. Pulsus paradoxus testing was not performed.

**Figure 1:**
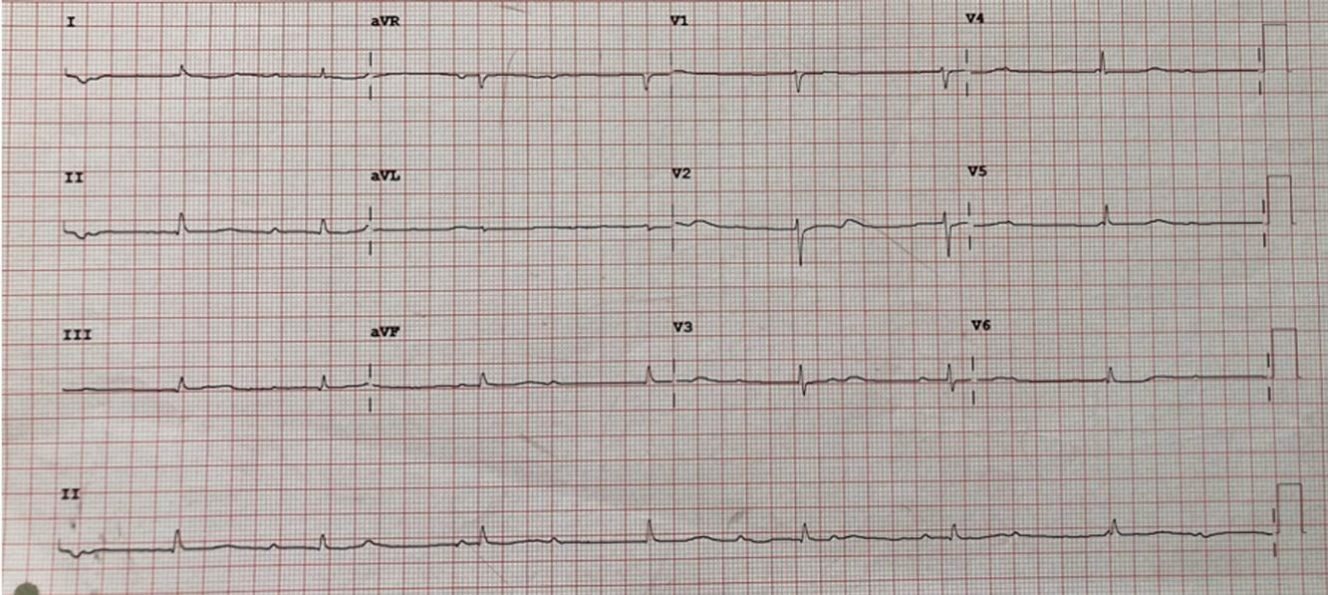
Third-degree heart block with small QRS complexes on electrocardiogram.

Electrocardiogram (ECG) on admission revealed a complete heart block with small QRS complexes (*Figure 1*). A CT pulmonary angiogram (CTPA) was negative for pulmonary embolism but demonstrated a large pericardial effusion (>3 cm depth) with small to moderate bilateral pleural effusions and two enlarged mediastinal lymph nodes.

**Figure 2:**
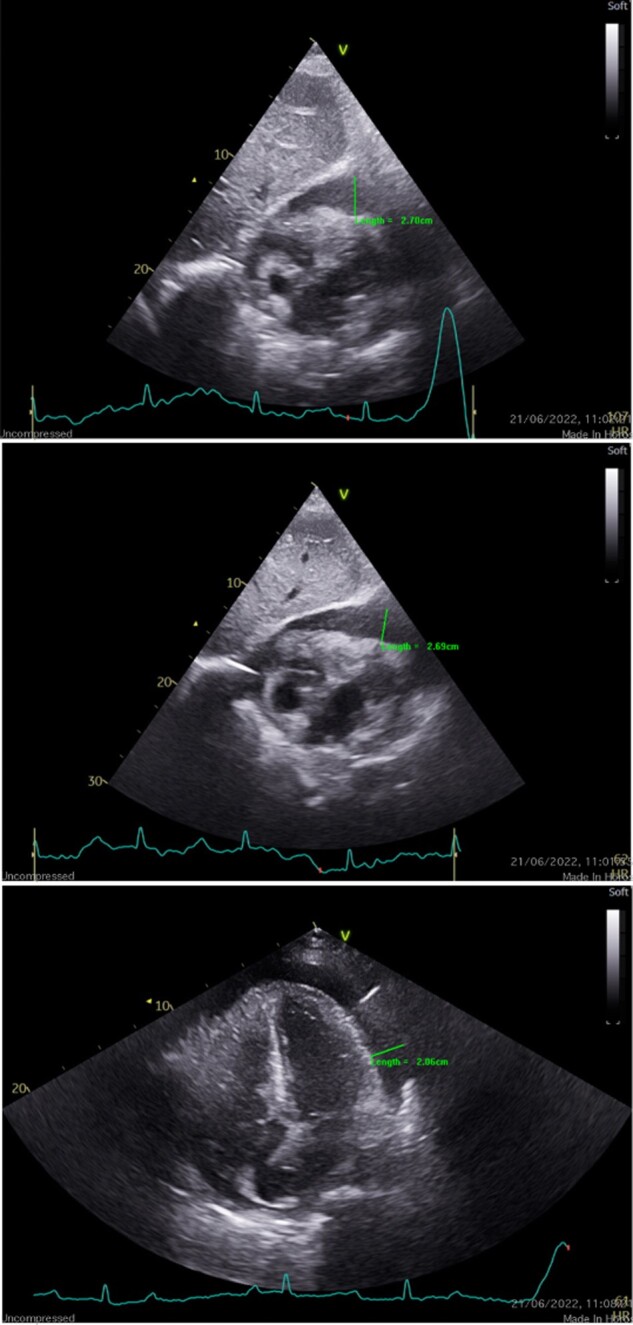
Transthoracic echo shows a large circumferential pericardial effusion measuring 2.7 cm at its largest, with evidence of right chamber collapse.

Transthoracic echocardiography (TTE) demonstrated normal left ventricular systolic function with an estimated left ventricular ejection fraction of 60%. No significant valvular abnormalities were seen. A large circumferential pericardial effusion measuring 27 mm with evidence of right ventricular and right atrial diastolic collapse was present (*Figure 2*). The patient’s full blood count was normal. Her renal profile revealed Creatinine of 96 µmol/L (49–90). Neither is consistent with tumour lysis syndrome (TLS). High Sensitivity troponin was 108 ng/L (<16) and an elevated brain natriuretic peptide of 464.8 ng/L (<100) was noted.

The patient’s condition deteriorated early in her admission. Cardiac tamponade was confirmed clinically. Marked haemodynamic instability was present with a blood pressure measurement of 88/46 mmHg and bradycardia reaching a maximum of 48 bpm. Emergency pericardiocentesis was successfully performed draining a total of 700 mL of blood-tinged serosanguinous fluid from the pericardial space. A dual chamber permanent pacemaker (PPM) was then implanted for treatment of complete heart block. Following these interventions, the patient’s blood pressure normalized at 127/60 mmHg, and heart rate was recorded at 70 bpm reflecting a paced rhythm.

Initial cytological analysis of the pericardial fluid was negative for malignant cells. Mesothelial cells, macrophages, and B and T cell lymphocytes were noted in the sample. Elevated levels of lactate dehydrogenase (LDH) (1955IU/L) were noted.

**Figure 3:**
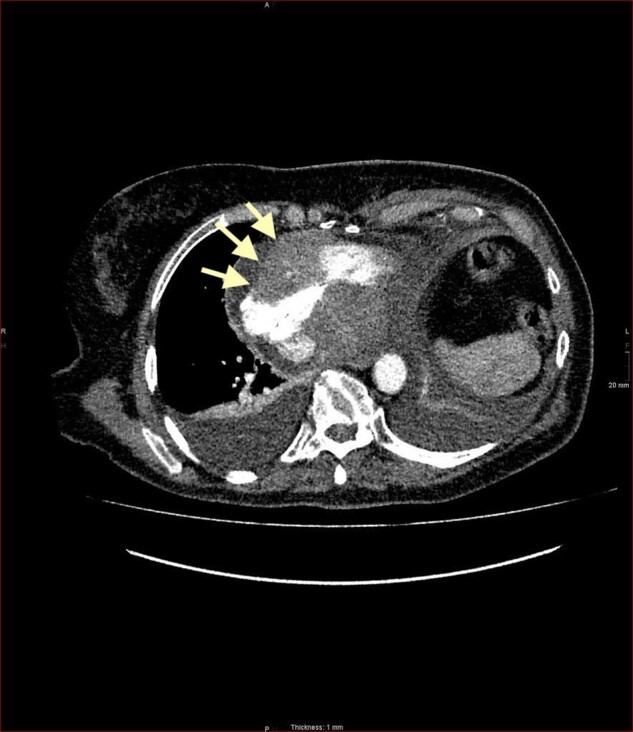
Lobulated soft tissue mass extending into the musculature of the right atrium and bilateral pleural effusions on computed tomography thorax.

**Figure 4:**
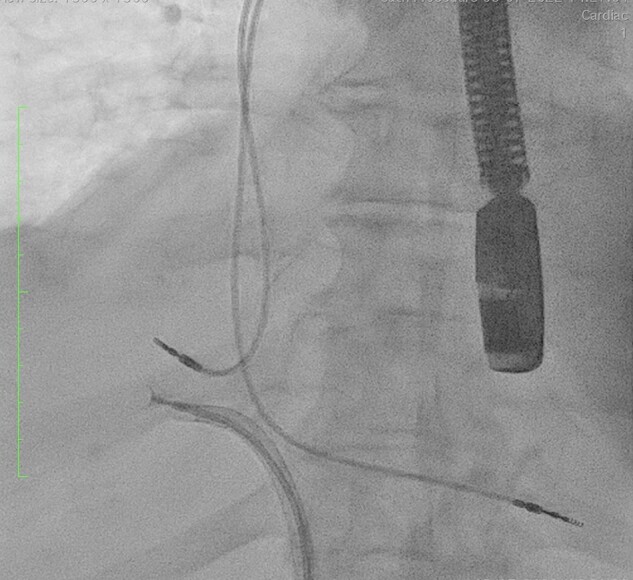
Transoesophageal echocardiogram and fluoroscopy-guided endomyocardial biopsy of cardiac mass. A steerable Agilis catheter is used to position the bioptome at the anterior right atrial wall to obtain tissue sample.

**Figure 5:**
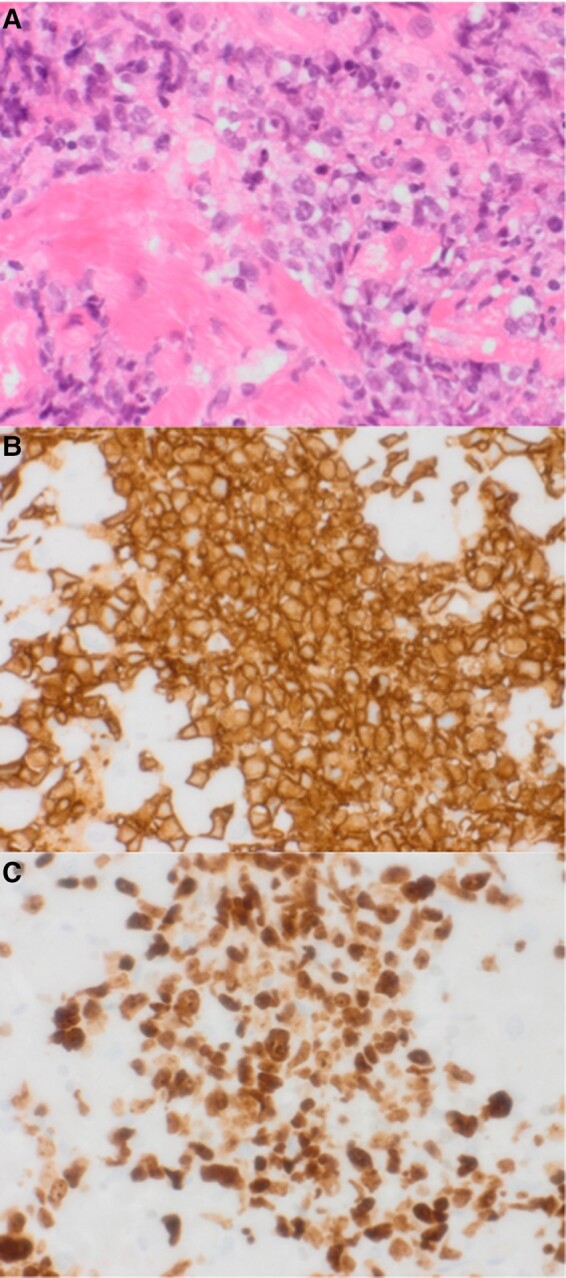
Histology and immunohistology of myocardial tissue: (*A*) Histology shows myocardial tissue infiltrated by pleomorphic medium to large-sized lymphoid cells with round vesicular nuclei and prominent nucleoli (haematoxylin and eosin staining; ×40). (*B*) Immunohistochemistry shows tumour cells strongly positive for CD20 (× 40). (*C*) Immunohistochemistry using Ki-67 shows a high proliferation index of ∼70% (× 40).

A post drainage CT thorax abdomen and pelvis (CTTAP) with contrast was completed which showed a lobulated soft tissue mass surrounding the right coronary artery extending transmurally involving the atrioventricular junction to encroach into the right atrium. It measured 52 mm at its maximum diameter. (*Figure 3*). These findings were suggestive of cardiac neoplasm and the differential diagnosis included lymphoma and sarcoma. The patient underwent a transoesophageal echocardiogram (TOE) guided endomyocardial biopsy. The initial approach for the biopsy used a standard 7 French catheter via transfemoral access. Steering the catheter tip to the required location on the anterior wall of the right atrium proved technically challenging. Therefore, an Agilis steerable sheath was used which allowed optimal positioning of the bioptome and biopsy of the right atrial mass (*Figure 4*). Histology of the tissue samples confirmed a diffuse large B-cell lymphoma ABC subtype (Non-Hodgkin’s lymphoma) (*Figure 5*).

A multidisciplinary team approach, with collaboration between cardiology, medical oncology, and pathology teams, was employed to formulate a management plan.

Following considerations including patient age, co-morbidities, disease progression, and prognosis, chemotherapy with rituximab, cyclophosphamide, hydroxydaunorubicin, oncovin, and prednisolone (R-CHOP) was deemed to be the most appropriate treatment. The patient declined all further intervention, opting instead for conservative symptomatic management of her disease progression. The patient died from her disease 1 month later.

## Discussion

Cardiac conduction issues paired with tamponade in a patient with no history of cardiac disease have broad differential diagnoses. In addition to cardiovascular disease, rare infiltrating diseases of infectious (e.g. tuberculosis) or malignant aetiology should be considered.^6^ It is of particular importance to include the latter differential in an aging population.^7^ Determining the primary cause of our patient’s presentation was guided by the discovery of a cardiac mass following drainage of pericardial effusion.

Cardiac tumours presenting with complete heart block are reported in the literature.^2,8–10^ However, typically dyspnoea and cardiac congestive symptoms are considered more common presentations in the setting of infiltrative cardiac tumours.^8^ In a number of cases where cardiac lymphoma has precipitated complete heart block, treatment of the lymphoma, either medically or surgically, has resulted in improved dysrhythmia or a reduced pacing burden in those with pacemakers.^2,8^

Although it is recognized that most cardiac masses are benign, and the pericardial fluid analysis, in this case, was negative for malignant cells, elevated LDH levels did raise suspicion for malignant aetiology. Further workup with imaging and biopsy allowed confirmation of a malignant cardiac tumour. The reported rates of false negative cytology from pericardial fluid range widely in the literature (1.7%–34%).^11–13^ Interestingly, haematological malignancies such as lymphoma typically have a higher incidence of false negative malignant pericardial fluid cytology.^13^ Overall, research suggests diagnosis should not be based solely on pericardial fluid cytology.

The use of multimodality imaging in the investigation of a cardiac mass can be invaluable. It allows for characterization of the mass and subsequently allows for narrowing of differential diagnoses.^14^ TTE, TOE, CT, CMR, and PET are all modalities recommended in the ESC 2022 cardio-oncology guidelines for the assessment of cardiac masses.^5^ Our patient underwent CTPA and TTE confirming initial cardiac tamponade. Following pericardiocentesis and PPM implantation, a CT Thorax identified and characterized the mass and its location within the heart. Pacemaker implantation had been prioritized due to the presence of persistent symptomatic complete heart block. Unfortunately, due to the close time proximity to pacemaker insertion, this precluded CMR from being performed.

Furthermore, the ESC guidelines state tissue biopsy and cytology are the gold standard for establishing a diagnosis of aggressive cardiac malignancies such as PCL.^5^ The appropriate biopsy technique is best determined by consideration of the characteristics of a particular tumour (e.g. size and location).^15^ Some institutions choose thoracotomy for biopsy acquisition, but less invasive percutaneous approaches are favoured when feasible.^15,16^ In this case, consensus involving the Cardiology and Interventional Radiology team at our institution favoured sample acquisition via percutaneous endovascular approach. Obtaining endomyocardial biopsies via percutaneous techniques requires a range of technical considerations. One such consideration is achieving optimal catheter placement for tissue sampling, particularly for cardiac tumours located in challenging positions of the heart. Originally developed to facilitate cardiac ablation procedures, steerable sheaths offer increasing ease of accessibility to sites of interest for a range of cardiac procedures. Due to the location of our patient’s cardiac mass, within the anterior right atrial wall, biopsy was technically challenging. A standard introducer sheath was sub-optimal for positioning the bioptome. Therefore, an Agilis steerable sheath was introduced to allow a precise biopsy of the desired region.

Owing to the rarity of PCLs, there is a lack of specific management guidelines. Consensus from individual case reports suggests that the main approach to management is systemic chemotherapy.^17^ Most commonly combination chemotherapy with R-CHOP regimen is used. Timely treatment of PCL with this chemotherapeutic approach has demonstrated beneficial responses with reports of disease remission, resolution of precipitated cardiac complications such as obstructive symptoms or arrhythmias, and survival benefit. Retrospective analysis of a large American database has shown a significant improvement in 5-year overall survival from 38.1% in the 2003–2006 cohort to 53.1% in the 2013–2016 cohort probably due to the widespread adoption of Rituximab in combination with CHOP.^18^ This database also showed that age and co-morbidities were adverse prognostic factors, and the single best predictor of survival was receiving chemotherapy. Less frequently management with surgical resection or radiation therapy, either radical or adjuvant therapy, is used, although prognosis is poor in these groups.^17^ Specifically, in cases where PCL has presented with cardiac rhythm disturbances, early management of the tumour leads to improvements in cardiac symptoms. Decreased pacing burden in patients with pacemakers is reported following immunochemotherapy treatment. A small number of case reports document outright reversal of complete heart block or other arrhythmias following treatment of tumours.^2,8–10^ These cases demonstrate that early appropriate treatment of this highly sensitive cardiac tumour can improve the precipitated cardiac conduction sequalae.

## Conclusion

Although rare, clinicians should be open to the possibility of cardiac tumours, such as PCL, presenting with cardiac tamponade and cardiac arrhythmia. Multimodality imaging and tissue biopsy are required in the diagnostic workup of PCL, as specified by ESC guidelines. Prompt diagnosis and treatment can influence outcomes favourably.

## Supplementary Material

ytad635_Supplementary_Data

## Data Availability

The data underlying this article are available in the article and in its online Supplementary material.

## Lead author biography

Ruth Kelleher is a doctor in training from Ireland. She currently works at Cork University Hospital as a registrar in radiation oncology. Her areas of interest are radiology, including cardiac imaging, oncological radiology, and medical education.

## Supplementary material

**Consent:** The authors of this report confirm that written informed consent for submission and publication of this case report including images and associated text has been obtained from the patient in line with COPE guidance.

**Funding:** None declared.
