# Supplementary figures and images for: Primary cardiac lymphoma presenting with cardiac tamponade and complete heart block: case report
Source: Eur Heart J Case Rep. 2023 Dec 23;8(1):ytad635. doi: 10.1093/ehjcr/ytad635 (PMC10787369; doi:10.1093/ehjcr/ytad635)

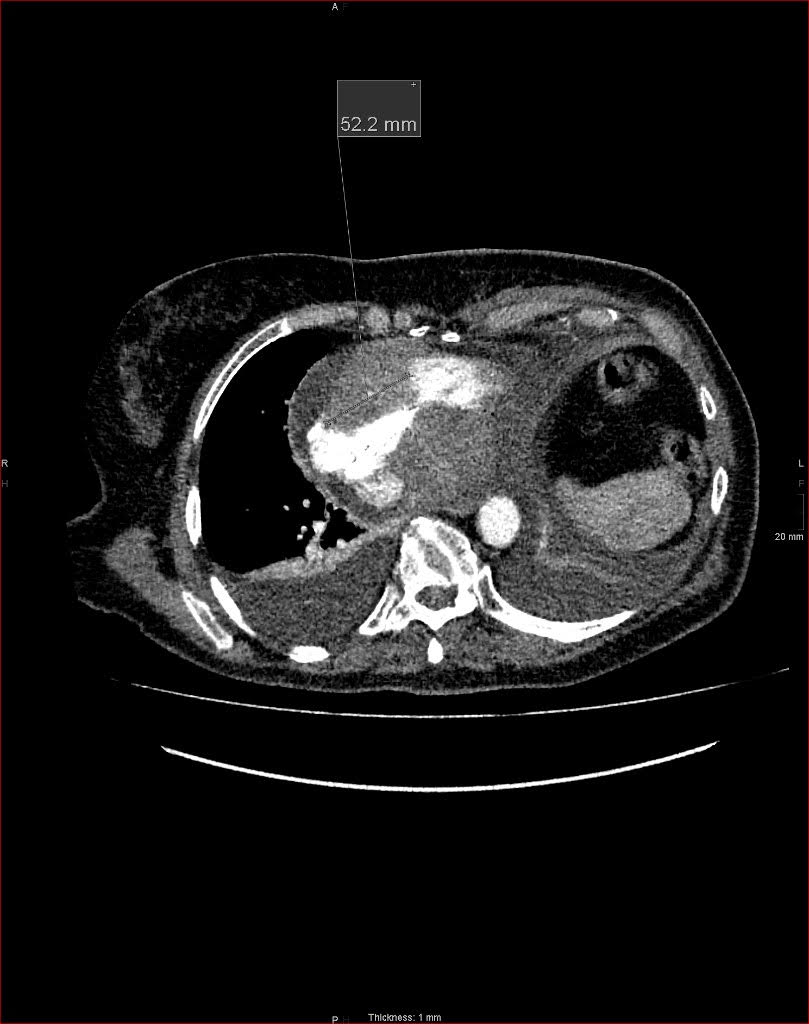

Supplement: ytad635_Supplementary_Data [file ytad635_supplementary_data.zip › CT Thorax Cardiac Mass max dimensions.jpg]

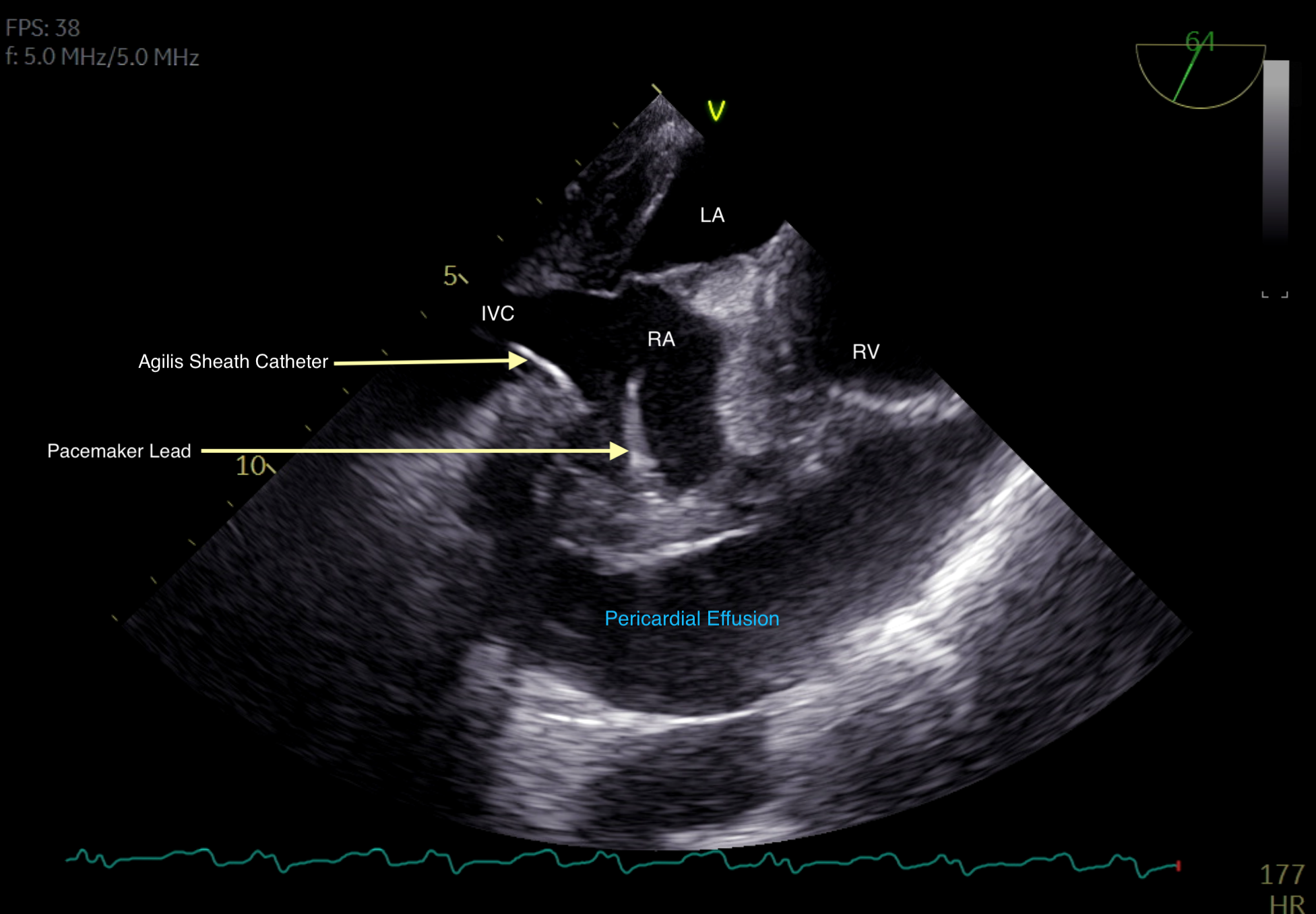

Supplement: ytad635_Supplementary_Data [file ytad635_supplementary_data.zip › Still only TOE biopsy Image .tif]
